# Intraguild Predation Among Three Thrips Species and the Two-Spotted Spider Mite *Tetranychus urticae* on Vegetables

**DOI:** 10.3390/insects17080873

**Published:** 2026-08-21

**Authors:** Bo Peng, Jin-Cui Chen, Ming Xue, Bing Li, Alima Azhati, Ya-Jun Gong, Yan-Li Du, Shu-Jun Wei

**Affiliations:** 1 College of Plant Science and Technology, Beijing University of Agriculture, Beijing 102206, China15199521979@163.com (A.A.); 2 Institute of Plant Protection, Beijing Academy of Agriculture and Forestry Sciences, Beijing 100097, Chinaxueming0702@163.com (M.X.);

**Keywords:** *Frankliniella occidentalis*, *Thrips palmi*, *Megalurothrips usitatus*, *Tetranychus urticae*, intraguild predation, omnivory, qPCR

## Abstract

Several phytophagous thrips can act as facultative predators within arthropod food webs, and this trophic flexibility may shape pest population dynamics when thrips co-occur with spider mites or conspecific competitors on shared host plants. Here we examined intraguild predation among three economically important thrips species, *Frankliniella occidentalis*, *Thrips palmi*, and *Megalurothrips usitatus*, and the two-spotted spider mite *Tetranychus urticae*, which frequently cooccur on vegetable crops. Predation assays showed that all three thrips species consumed *T. urticae*, but predation varied markedly with predator species, predator stage, and prey stage. Mite eggs were the most vulnerable prey stage with *F. occidentalis* showing high egg predation across developmental stages, while *T. palmi* and *M. usitatus* showed lower and more variable predation. Predation on active mite stages was generally weaker. Intraguild interactions among the three thrips species were limited in active-stage assays, but larval *M. usitatus* significantly reduced the survival of *F. occidentalis* eggs. Removing host plant material increased predation on *T. urticae* eggs, especially for *F. occidentalis* and *T. palmi*. In 20-day mixed-rearing assays, co-rearing with thrips significantly reduced active *T. urticae* abundance, whereas pairwise thrips co-rearing produced no clear suppression of thrips population abundance. Species-specific qPCR confirmed prey DNA in a subset of predators after feeding assays, supporting the direct trophic interactions detected in the behavioral experiments. These findings highlight the need to treat thrips not only as herbivores but also as context-dependent omnivores when evaluating pest interactions and integrated pest management strategies.

## Simple Summary

Thrips and spider mites are among the most damaging small arthropod pests of vegetable crops, and they frequently share the same host plants. In addition to feeding on plants, several thrips species can also feed on other small arthropods, but how common this behaviour is among vegetable-pest thrips, and whether it affects mite populations or other thrips, is not well understood. In this study we tested whether three common vegetable thrips species can prey on two-spotted spider mites and on each other, and how the presence of a host plant changes their predatory behaviour. We combined behavioural experiments in small arenas, a longer 20-day mixed-rearing test, and a molecular assay that detects prey DNA inside individual thrips. All three thrips species fed on mite eggs, especially when no plant tissue was present, but had little effect on each other, except that one species reduced the survival of another species’ eggs. Over 20 days, thrips reduced mite numbers but did not clearly suppress other thrips. These results show that vegetable thrips can act as facultative predators of spider mites and should be considered when planning integrated pest management.

## 1. Introduction

Thrips (Thysanoptera: Thripidae) and tetranychid spider mites (Acari: Tetranychidae) are economically important arthropod pests [1,2]. The invasive western flower thrips *Frankliniella occidentalis* (Pergande), melon thrips *Thrips palmi* Karny, and bean flower thrips *Megalurothrips usitatus* (Bagnall) are among the most significant thrips pests [3,4,5,6], while the two-spotted spider mite *Tetranychus urticae* Koch is an extremely polyphagous mite pest [7,8]. Multiple thrips species and spider mites may co-occur on the same host plant, potentially leading to interspecific competition and complex ecological interactions [9].

Omnivory, the consumption of resources from more than one trophic level, is widespread among thrips and has been documented for numerous species [10,11]. *Frankliniella occidentalis*, in particular, is known to feed opportunistically on spider mite eggs and has been shown to derive fitness benefits from such mixed diets [12,13]. On Australian cotton, *Thrips imaginis* Bagnall, *T. tabaci* Lindeman, and *Frankliniella schultzei* (Trybom) consumed *T. urticae* eggs in laboratory assays and exhibited field preferences for spider-mite-infested plants, suggesting a potential role in early-season mite suppression [13,14]. Specialist predatory thrips such as *Scolothrips longicornis* Priesner can consume large numbers of *T. urticae* eggs under laboratory conditions [15,16]. A recent synthesis identified approximately 100 thrips species across Aeolothripidae, Phlaeothripidae, and Thripidae with predatory behaviour, though quantitative consumption data remain sparse for most species [11].

When thrips co-occur, they may prey on one another while also competing for shared resources, a combination of competition and predation termed intraguild predation (IGP), which can affect community stability, predator effectiveness, and species coexistence [17,18,19]. IGP is ecologically important because it alters the dynamics of competition, coexistence, and biological control [17,19]. In thrips-dominated vegetable systems, multiple pest species may interact directly while also exploiting shared host plants, creating complex food-web dynamics that are not captured by treating each pest in isolation [20,21].

Less is known about whether common vegetable pest thrips can suppress spider mites or prey on heterospecific thrips competitors through facultative predation [11,12,13]. Several studies have noted that mite egg predation by thrips can make a disproportionate contribution to mite suppression, as eggs are stationary, lack escape responses, and are encountered at high densities on plant surfaces where thrips forage [12,13].

Addressing these gaps requires both controlled behavioral assays to quantify predation, as well as examination of host-plant context effects on predation outcomes. DNA-based gut-content analysis and qPCR have become valuable tools for verifying predator–prey interactions in arthropods, although positive detections must be interpreted cautiously, as prey DNA can sometimes be acquired through indirect pathways such as consumption of prey-contaminated honeydew or secondary predation [22,23,24,25,26]. Combining controlled behavioral assays with species-specific molecular validation therefore provides a stronger basis for evaluating trophic interactions among small arthropods [27]. Host-plant conditions also influence predation rates, as plant tissue can reduce encounter probability between predators and prey, provide structural refuges for mite eggs, or supply alternative food resources that reduce reliance on animal prey [28,29,30].

The present study examined predation and intraguild interactions involving three vegetable thrips species, *F. occidentalis*, *T. palmi*, and *M. usitatus*, and the two-spotted spider mite *T. urticae*, with the following objectives: (i) to compare predation by different thrips species and developmental stages on eggs, nymphs, and adults of *T. urticae*; (ii) to assess intraguild predation among laboratory populations of the three thrips species; (iii) to determine whether host-plant substrate affects predation rates and longer-term population outcomes under pairwise mixed rearing.

## 2. Materials and Methods

### 2.1. Thrips and Mites

Three thrips species (*M. usitatus*, *T. palmi*, and *F. occidentalis*) and the spider mite *T. urticae* were used in this study. The *M. usitatus* colony was established from a population collected from Hainan Province, China, on cowpea (*Vigna unguiculata*); *T. palmi*, *F. occidentalis*, and *T. urticae* were established from populations collected from the Beijing area, China, from eggplant, pepper, and strawberry, respectively. All colonies had been maintained as laboratory populations for two to five generations prior to the experiments. Thrips were reared in glass jars covered with fine mesh and supplied with fresh sword bean (*Canavalia gladiata*) pods as a food source and oviposition substrate. *Thrips palmi* was instead reared with cucumber (*Cucumis sativus*) seedlings as the oviposition substrate. *Tetranychus urticae* was maintained on cowpea (*Vigna unguiculata*) seedlings in insect-rearing cages. All colonies were maintained in a climate-controlled rearing room at 25 °C, 50 ± 10% relative humidity (RH), and a 16 h:8 h, light: dark photoperiod. First- and second-instar larvae were distinguished under Stemi 305 stereomicroscope (Carl Zeiss Microscopy GmbH, Jena, Germany) based on relative body size and instar-specific larval morphology, with first-instar larvae being distinctly smaller than second-instar larvae. First-instar larvae, second-instar larvae, and adults of each thrips species were used as both prey and predators. Eggs, nymphs, and adults of *T. urticae* were used as prey in predation assays.

### 2.2. Experimental Arenas

Behavioral assays were mainly conducted in covered cylindrical containers (bottom diameter 53 mm, height 43 mm, approximate volume 94.86 cm^3^). A 3 mm layer of 2% agar was applied to the base of each arena to maintain plant freshness; plant material was placed on the agar surface and sealed to reduce desiccation and prevent escape. The population mixed-rearing experiment was conducted in cylindrical glass containers (bottom diameter 9 cm, height 17 cm, approximate volume 1081.7 cm^3^). All experiments were conducted at 25 °C, 50 ± 10% RH, and a 16 h:8 h, light: dark photoperiod. For each assay, 10 treatment arenas and three control arenas were initially established, unless otherwise specified. Replicates were excluded from analysis when escape of test individuals, failure of arena integrity or maintenance, severe plant desiccation, fungal contamination, or abnormal mortality occurred. Consequently, the final number of valid biological replicates varied among treatments.

### 2.3. Predation of T. urticae by Three Thrips Species

To compare predation by *M. usitatus*, *T. palmi*, and *F. occidentalis* on different developmental stages of *T. urticae*, first-instar larvae, second-instar larvae, and adults of each thrips species were paired with eggs, nymphs, and adults of *T. urticae*, producing 27 predator–prey combinations. For each replicate, 30 eggs or individuals of *T. urticae* were introduced into an arena, followed by 10 thrips of the corresponding species and developmental stage. Adult females were used to standardize the adult predator category across treatments and to minimize potential variation associated with sex; the experiment was not designed to compare sex-specific predation. Sex was not determined for immature stages. Each treatment was replicated ten times. Control arenas (three replicates each) contained *T. urticae* but no thrips. All treatments were assessed at 24 h.

Predation was expressed as the corrected predation rate (Abbott/Schneider–Orelli type formula):

P_corrected_ = 1 − (N_t_/N_0_)/(C_t_/C_0_) (1)

where N_0_ is the initial prey count in the predator treatment, N_t_ the surviving prey count after the assay, C_0_ the initial prey count in the control, and C_t_ the surviving prey count in the control.

### 2.4. Predation Among Three Thrips Species

For active-stage interactions, adults and second-instar larvae of the three thrips species were paired with heterospecific adults or second-instar larvae as intraguild prey (30 prey and 10 predators per arena; surviving prey recorded after 24 h; ten replicates per treatment; three control replicates). For heterospecific egg predation assays, 50 adult females of the prey species were allowed to oviposit on sword bean pods or leaf substrates for 48 h, then removed; 20 predators were introduced for 24 h, then removed; arenas were incubated for a further 72 h for counting the number of emerged first-instar larvae. This incubation period was selected based on preliminary observations of thrips egg development under 25 °C, during which hatching generally begins after 3–4 days, allowing newly emerged larvae to be used as a proxy for egg survival within a standardized observation window. Because thrips eggs are inserted into plant tissue, the number of newly hatched larvae was used as an indirect estimate of egg survival. Ten treatment arenas and three control arenas were initially established for each predator–prey combination.

### 2.5. Effects of Host Plant on Predation

Host-plant effects were examined using three substrate treatments (sword bean pod, cucumber leaf, and no host plant) for adult mite predation, and two substrate treatments (cowpea leaf and no host plant) for mite egg predation. Arenas received 30 mite eggs or adults and 10 adult thrips (five replicates per treatment; three control replicates).

### 2.6. Population Mixed-Rearing Experiment

To evaluate longer-term population-level interactions, pairwise mixed-rearing experiments were conducted over 20 days. In single-species controls, 20 active adults per species were introduced per container (10 replicates per species). Six mixed treatments were established (each of the three thrips species × *T. urticae*, plus all three pairwise thrips combinations), with 20 active adults per species per container (40 total; 10 replicates). One fresh sword bean pod (~20 cm) was initially provided per container; an additional pod was added every 72 h. After 20 days, visible active individuals (nymphs and adults) were counted; adults in mixed thrips treatments were identified to species by morphological characteristics.

### 2.7. Molecular Validation by qPCR

Predator–prey combinations with comparatively high corrected predation rates or significant predation outcomes in behavioral assays were selected for molecular validation by quantitative real-time PCR. Selected combinations included adult *F. occidentalis* feeding on *T. urticae* eggs, adult *T. palmi* feeding on *T. urticae* eggs, adult *M. usitatus* feeding on *T. urticae* eggs, adult *T. palmi* feeding on *T. urticae* nymphs, adult *F. occidentalis* feeding on second-instar larvae of *M. usitatus*, and second-instar larvae of *M. usitatus* feeding on eggs of *F. occidentalis*.

After predation assays, each predator individual was collected singly, immobilized by freezing in liquid nitrogen, and DNA was extracted by a rapid lysis method (65 °C, 1 h; 95 °C, 10 min). At least 20 predator individuals were tested per combination. Species-specific primers (Table 1) were used to detect DNA from *T. urticae*, *F. occidentalis*, *T. palmi*, and *M. usitatus* [31,32,33]. Positive controls, negative controls, no-template controls, and predator DNA quality controls were included in each run. qPCR was performed using TB Green Premix Ex Taq II (Tli RNaseH Plus) (Kusatsu, Japan) on an Applied Biosystems QuantStudio 7 Flex Real-Time PCR System (Foster City, CA, USA) (25 µL reaction; 95 °C for 10 min, then 40 cycles of 95 °C for 15 s and 60 °C for 60 s; followed by melting curve analysis). A sample was considered positive only when it showed a stable amplification curve and a melting peak consistent with the positive control; ambiguous or discordant samples were excluded [22,23,24]. In total, 21 or 32 individual predators were tested by qPCR.

Detection rate was calculated as:

Detection rate = (Number of positive predators/Total predators tested) × 100%(2)

### 2.8. Statistical Analysis

Two-group comparisons were performed using two-sided Mann–Whitney U tests. All statistical analyses were conducted using SPSS Statistics version 27 (IBM Corp., Armonk, NY, USA). Data are presented as means ± standard errors, with individual biological replicates shown in the figures. Statistical significance was set at p<0.05.

## 3. Results

### 3.1. Predation on T. urticae Varied by Thrips Species, Predator Stage, and Mite Prey Stage

**Figure 1:**
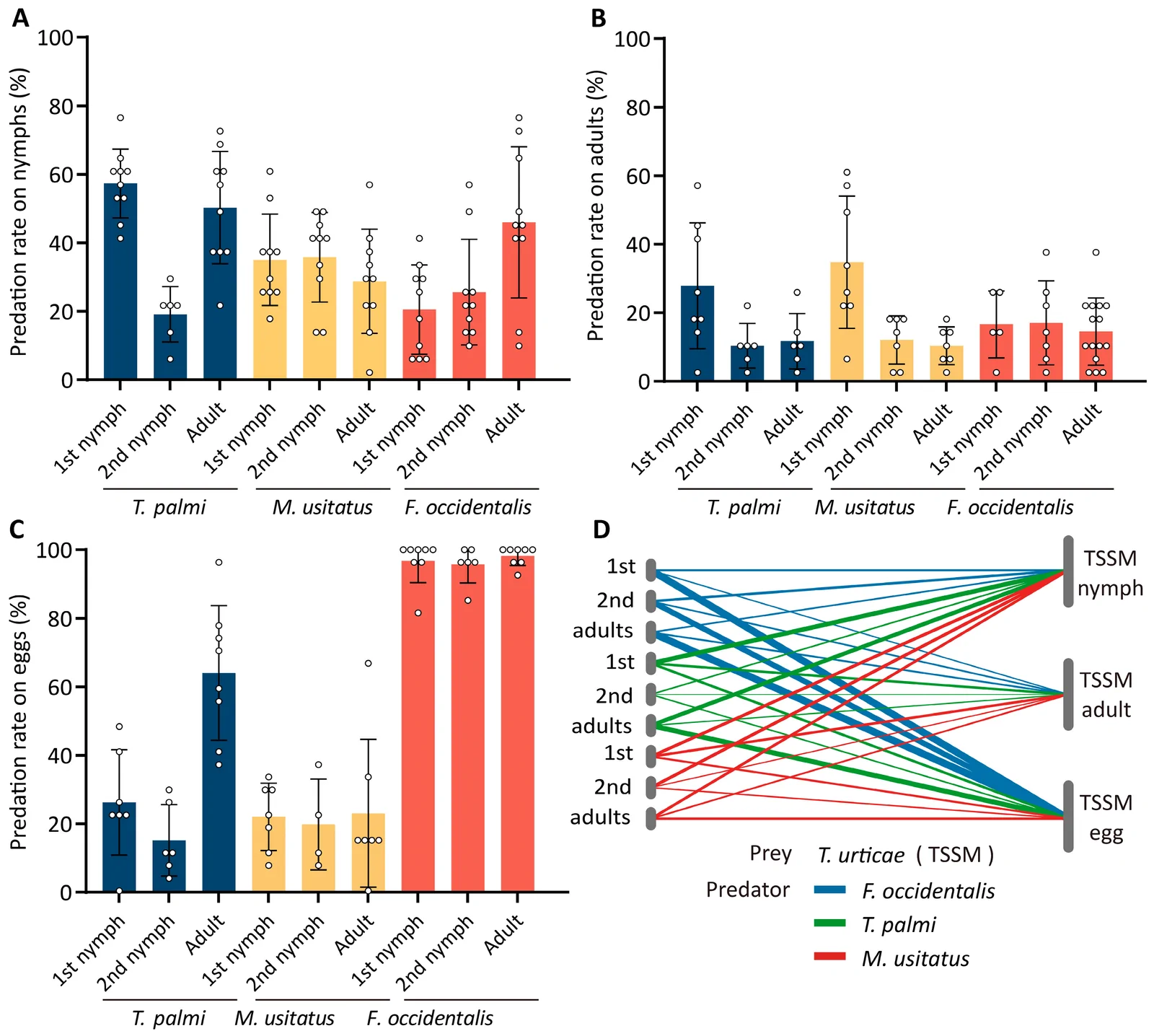
Predation of different developmental stages of *Tetranychus urticae* by three vegetable thrips species (*n* = 10). (**A**) Predation rate on mite nymphs. (**B**) Predation rate on adult mites. (**C**) Predation rate on mite eggs. (**D**) Layered interaction network summarizing predation links between thrips predators and *T. urticae* prey stages. Bars represent mean corrected predation rates ± SE; open circles indicate individual biological replicates. Line colors represent predator species; line width indicates relative predation effectiveness.

All three thrips species showed evidence of predation on *T. urticae*, but the magnitude and prey-stage specificity differed among species and developmental stages (Figure 1). Mite eggs were the most vulnerable *T. urticae* stage. *Frankliniella occidentalis* showed consistently high corrected predation rates on *T. urticae* eggs across first-instar (96.8 ± 2.3%), second-instar (95.7 ± 2.2%), and adult (98.2 ± 1.0%) predator stages. *Thrips palmi* showed stage-dependent egg predation, with adults (64.1 ± 7.0%) producing higher rates than first- and second-instar larvae (26.3 ± 5.8% and 15.2 ± 4.3%, respectively). *Megalurothrips usitatus* showed lower to moderate egg predation across stages (19.8 ± 6.6–23.1 ± 8.1%).

Predation on mite nymphs was generally moderate and variable. *Thrips palmi* first-instar larvae (57.3 ± 3.2%) and adults (50.3 ± 5.2%) showed comparatively higher nymph predation than the corresponding second-instar treatment (19.1 ± 3.3%). *Megalurothrips usitatus* showed moderate predation across stages (28.8 ± 4.8–35.8 ± 4.1%), while *F. occidentalis* showed lower nymph predation (20.5 ± 4.1–25.6 ± 4.9%) in early stages but higher predation by adults (46.0 ± 7.0%). Predation on adult *T. urticae* was generally lower than on mite eggs (10.4 ± 2.1–34.7 ± 6.8%) and inconsistent across predator stages. A layered interaction network summarizing all 27 combinations indicated strong links between *F. occidentalis* and mite eggs, moderate links involving selected *T. palmi* or *M. usitatus* stages and mite nymphs or eggs, and weaker links with adult mites (Figure 1D).

### 3.2. Active-Stage Intraguild Interactions Among Thrips Were Weak

**Figure 2:**
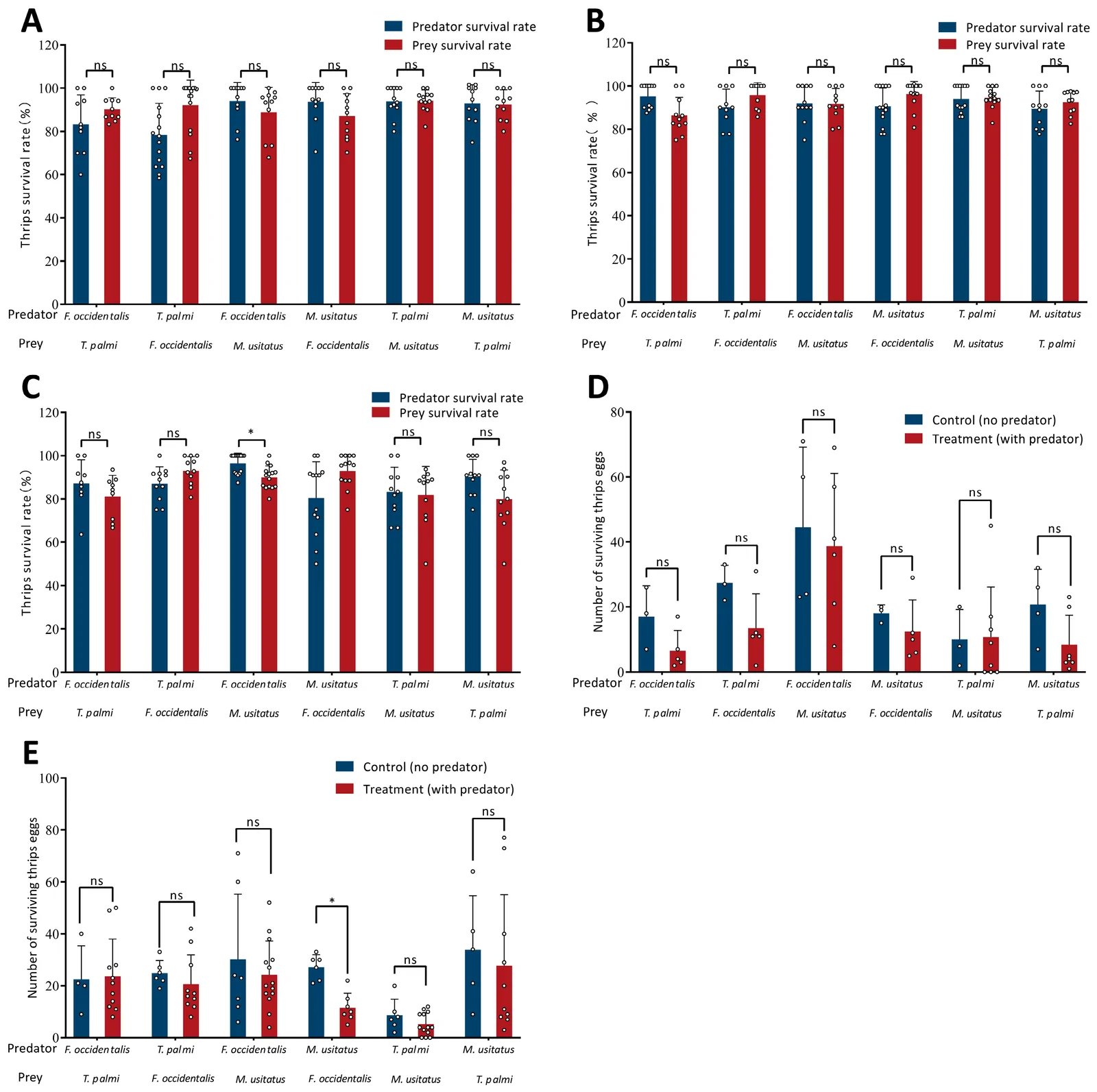
Intraguild interactions among three thrips species (*n* = 10 per treatment in (**A**–**C**,**E**); *n* = 4 per treatment in (**D**)). (**A**) Adult–adult interactions. (**B**) Adult predators with second-instar larval intraguild prey. (**C**) Second-instar larval predators with second-instar larval intraguild prey. (**D**) Predation by adult thrips on heterospecific eggs. (**E**) Predation by second-instar larvae on heterospecific eggs. Bars show means ± SE; open circles represent biological replicates. Blue and red bars represent predator and prey survival rates in panels (**A**–**C**), and control and predator-present treatments in panels (**D**,**E**). ns, not significant; * *p* < 0.01.

Adult–adult interactions among *F. occidentalis*, *T. palmi*, and *M. usitatus* did not substantially reduce survival of either partner compared with paired controls (Figure 2A), suggesting that direct adult-stage IGP was limited under 24-h assay conditions. When adult predators were paired with heterospecific second-instar larvae, survival remained high for both groups with no significant differences detected (Figure 2B). Second-instar larval predators paired with second-instar larval intraguild prey produced mostly non-significant effects (Figure 2C), with one exception: the interaction between *F. occidentalis* larvae and *M. usitatus* larvae showed a significant asymmetric survival difference.

### 3.3. Heterospecific Thrips Egg Predation Was Stage- and Species-Specific

Adult thrips did not significantly reduce heterospecific egg survival in the tested pairings (Figure 2D). In contrast, second-instar *M. usitatus* larvae significantly reduced survival of *F. occidentalis* eggs (57.4 ± 7.7%) (Figure 2E), whereas other larval predator × heterospecific egg combinations were not significant.

### 3.4. Host-Plant Context Modified Predation on T. urticae

**Figure 3:**
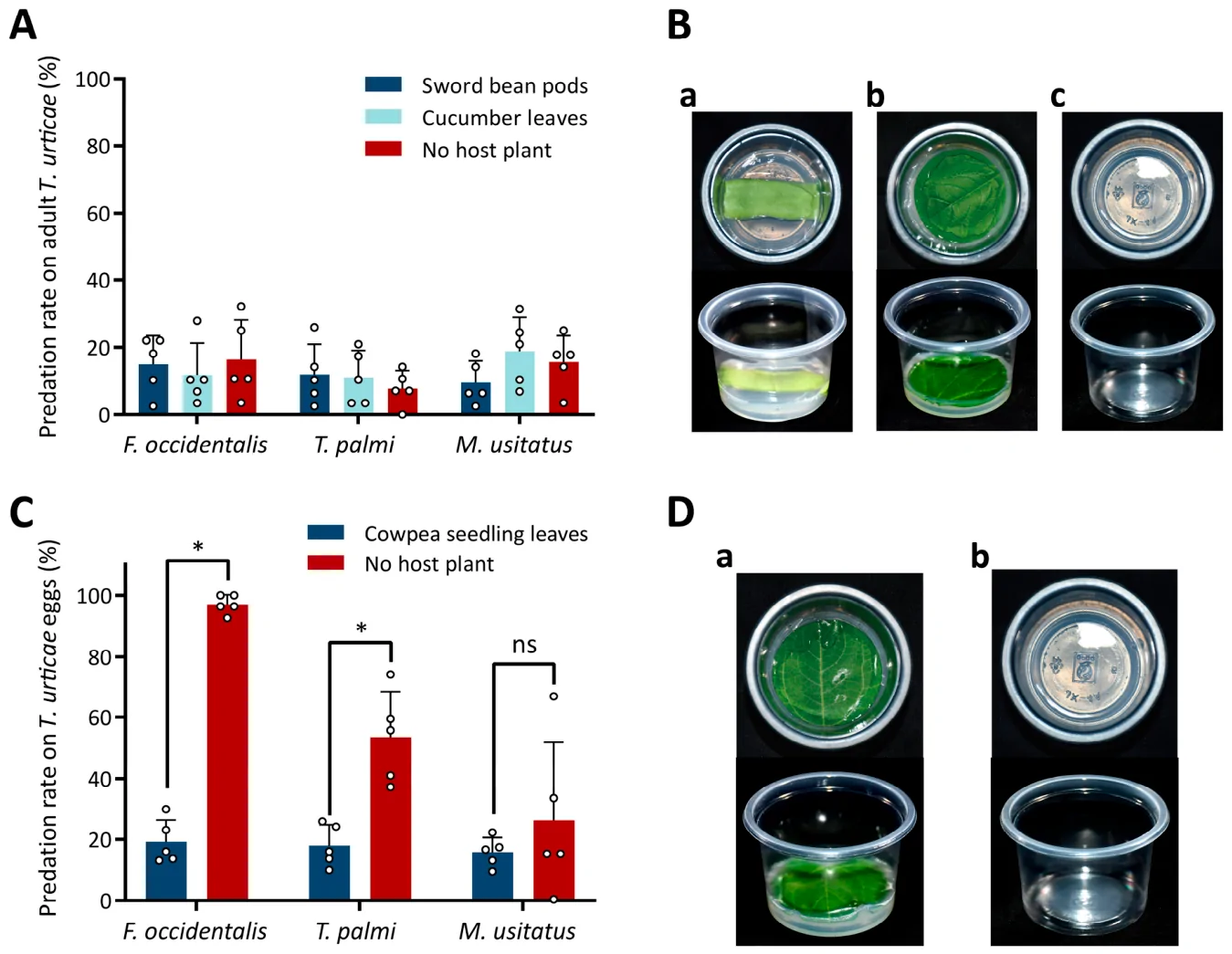
Effects of host plant on predation by thrips on *Tetranychus urticae* (*n* = 5). (**A**) Predation by adults of *F. occidentalis*, *T. palmi*, and *M. usitatus* on adult *T. urticae* under sword bean pod, cucumber leaf, and no host plant conditions. (**B**) Representative arenas for adult mite predation assays with sword bean pod (**a**), cucumber leaf (**b**), and no-host-plant conditions (**c**). (**C**) Predation by adults of the three thrips species on *T. urticae* eggs under cowpea-leaf and no host-plant conditions. (**D**) Representative arenas for mite egg predation assays with cowpea-leaf (**a**) and no host-plant conditions (**b**). Bars show means ± SE; open circles represent biological replicates. ns, not significant; * *p* < 0.01.

Host-plant conditions strongly altered predation by adult thrips on *T. urticae* eggs (Figure 3). Predation on adult mites remained relatively low across all substrate treatments for all three thrips species (Figure 3A). Predation on *T. urticae* eggs was strongly affected by host-plant context (Figure 3C). Under cowpea-leaf conditions, egg predation was comparatively low, while removing host-plant material markedly increased egg predation for *F. occidentalis* and *T. palmi*, with significant differences between plant and no-plant treatments. *Megalurothrips usitatus* showed a weaker and non-significant host-plant effect (Figure 3).

### 3.5. Pairwise Mixed Rearing Suppressed T. urticae but Not Heterospecific Thrips Populations

**Figure 4:**
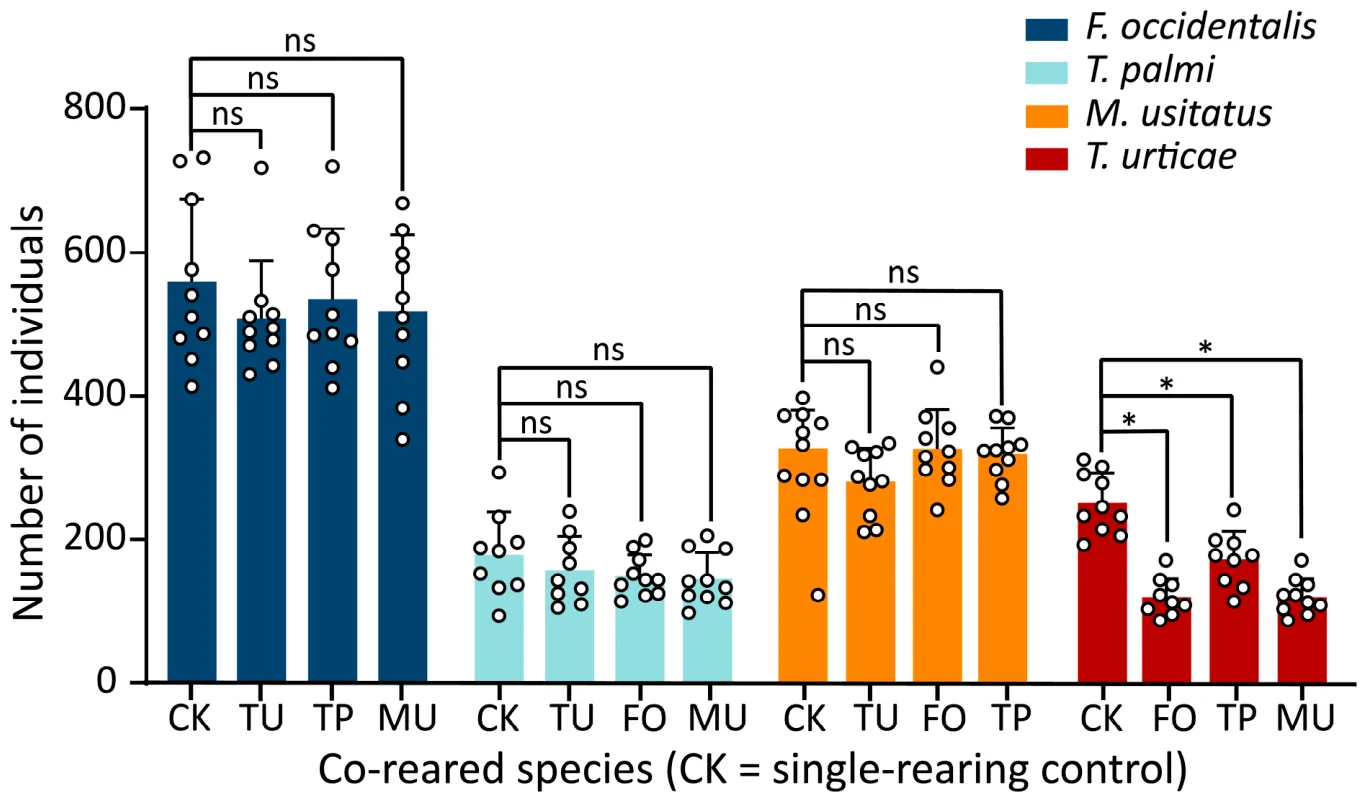
Effects of single and mixed rearing of thrips species and *Tetranychus urticae* on population growth (*n* = 10). Numbers of visible active individuals after 20 days in single-species controls and pairwise mixed cultures. CK, single-rearing control; TU, *T. urticae*; FO, *F. occidentalis*; TP, *T. palmi*; and MU, *M. usitatus*. Bars show means ± SE; open circles represent biological replicates. ns, not significant; * *p* < 0.01.

After 20 days of mixed rearing, thrips population abundance was not significantly affected by co-rearing with *T. urticae* or with another thrips species (Figure 4). In contrast, *T. urticae* abundance was significantly reduced when co-reared with each of the three thrips species (*M. usitatus*: 51.84 ± 3.17%; *T. palmi*: 31.08 ± 5.12%; *F. occidentalis*: 54.04 ± 3.33%), indicating that thrips can suppress visible active-stage mite abundance in mixed culture even though reciprocal effects on thrips populations were not detected.

### 3.6. qPCR Confirmed Prey DNA in Selected Predator–Prey Combinations

**Figure 5:**
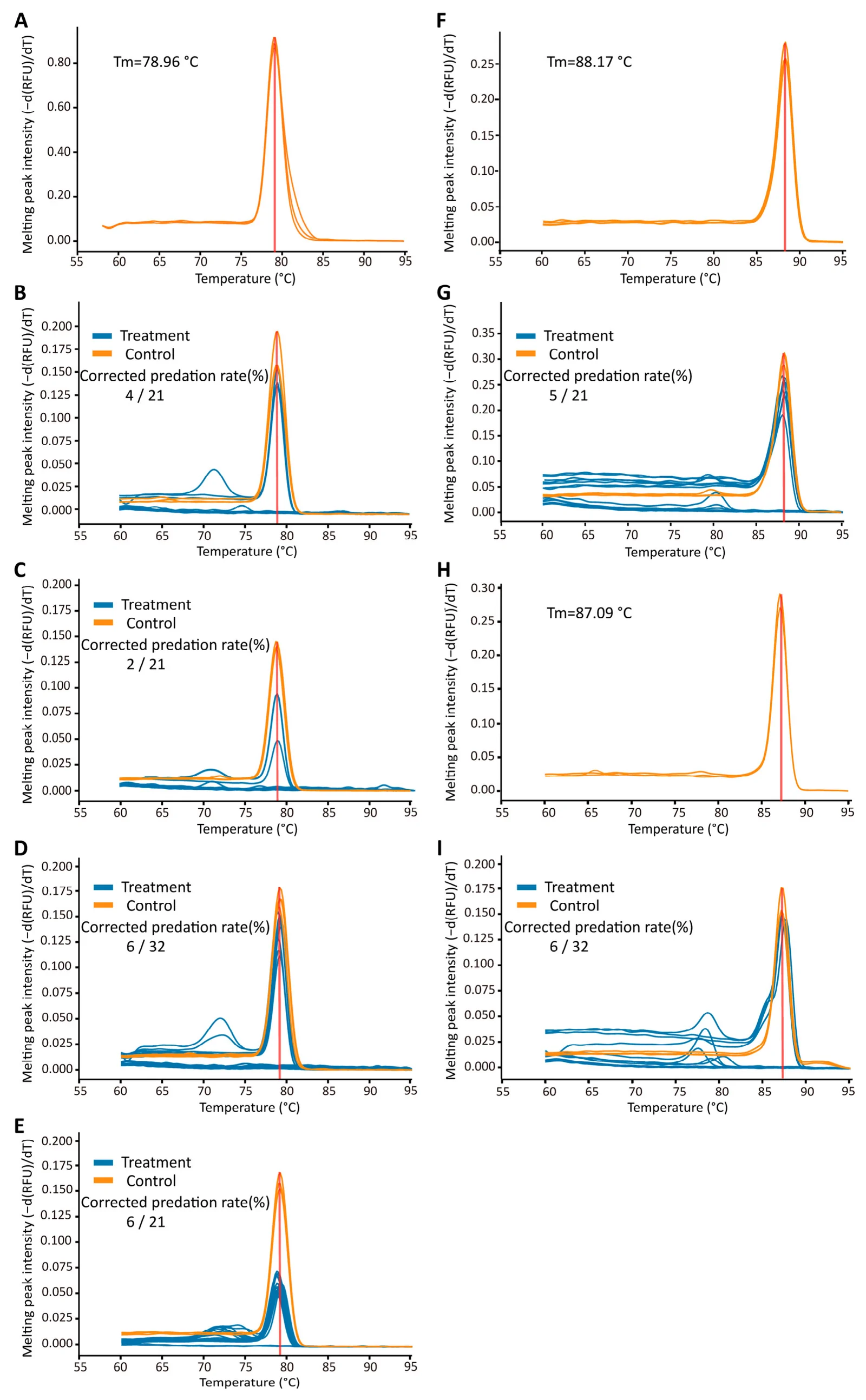
qPCR-based detection of prey DNA in thrips predators. (**A**) Melting peak of the *T. urticae* positive control. (**B**–**E**) Detection of *T. urticae* DNA in predators from selected mite predation assays. (**F**) Melting peak of the *F. occidentalis* positive control. (**G**) Detection of *F. occidentalis* DNA in *M. usitatus* larval predators fed on *F. occidentalis* eggs. (**H**) Melting peak of the *M. usitatus* positive control. (**I**) Detection of *M. usitatus* DNA in predators of *F. occidentalis* larva. Blue curves represent treatment samples, orange curves represent controls, and vertical red lines indicate target melting temperatures.

The *T. urticae* assay produced a target melting temperature of 78.96 °C, the *F. occidentalis* assay 88.17 °C, and the *M. usitatus* assay 87.09 °C (Figure 5). Prey DNA (*T. urticae*) was detected in 4/21 adult *F. occidentalis* feeding on mite eggs, 2/21 adult *T. palmi* feeding on mite eggs, 6/32 adult *M. usitatus* feeding on mite eggs, and 6/21 adult *T. palmi* feeding on mite nymphs. For intraguild combinations, prey DNA was detected in 5/21 predators in the *M. usitatus* larva × *F. occidentalis* egg assay, and 6/32 predators in the adult *F. occidentalis* × *M. usitatus* larva assay.

## 4. Discussion

This study demonstrates that three common vegetable thrips species can function as facultative predators of *T. urticae*, with the strongest predation directed toward mite eggs. *Frankliniella occidentalis* showed consistently strong predation on mite eggs, consistent with its documented role as an omnivorous opportunist that responds functionally to increases in spider mite egg density [12,13]. *Thrips palmi* showed stage-dependent predation with higher activity by adults on mite eggs and nymphs, while *M. usitatus* showed moderate but context-dependent predation. Considerable variation in predation rates was observed among replicates, potentially owing to variation in biological and experimental conditions and the stochastic nature of predator–prey encounters. Previous studies have documented omnivory and facultative predation in *F. occidentalis*, *T. imaginis*, *T. tabaci*, *F. schultzei*, and other thrips species [10,11,12,13,14,15,16]. Our findings extend this evidence by demonstrating and directly comparing predation on *T. urticae* by two additional economically important vegetable pests, *T. palmi* and *M. usitatus*.

The high vulnerability of mite eggs is biologically plausible [12,13,15]. Eggs are immobile, lack behavioral escape responses, and are encountered on plant surfaces where thrips forage. By contrast, mite nymphs and adults can move and respond to disturbance, making them more difficult to subdue. Even in obligate predatory thrips, similar prey-stage effects have been reported for *S. longicornis*, where egg predation makes a disproportionate contribution to total prey consumption, and for *F. occidentalis*, where functional response to mite egg density was demonstrated in cotton [15,16]. Previous work showed that spider mite webbing inhibited egg predation by *F. occidentalis* larvae [12]. In contrast, the arenas used in the present study contained no pre-established mite webbing, which may have contributed to the high observed rates of egg predation.

Host-plant context was a major determinant of observed predation [29,30]. Removing host-plant material strongly increased predation on mite eggs by *F. occidentalis* and *T. palmi*, while predation on adult mites remained low regardless of substrate. This result suggests that simplified arenas increase predator–prey encounter probability or reduce refuges for mite eggs, and also that plant tissue provides alternative feeding resources, reducing reliance on animal prey. This interpretation is consistent with predictions from food-web theory that when plant food is available, omnivores preferentially exploit plant resources, and predation on animal prey increases as plant food quality or availability declines [29,34]. In practical terms, predation rates measured in host-plant-free arenas represent a potential upper bound for predation capacity rather than realistic estimates under field crop conditions with intact plant architecture [28,30].

Active-stage intraguild interactions among the three thrips species were generally weak over 24 h, contrasting with the stronger effects observed against *T. urticae*. This is consistent with the general theoretical prediction that IGP intensity is higher when intraguild prey are less mobile or defensively equipped relative to the intraguild predator [18,21,35]. The occurrence of larval *M. usitatus* significantly reducing survival of *F. occidentalis* eggs indicates that egg-stage IGP can occur even when active-stage predation is limited. Because thrips eggs are inserted into plant tissue, such egg predation likely depends on predator probing behaviour, plant substrate structure, and the ability to detect concealed eggs. The broader IGP theoretical framework predicts that egg-stage interactions may have quantitative importance for population dynamics even when direct adult-stage predation is infrequent [17,19,21].

Population mixed-rearing experiments provided an important contrast to short-term arena assays. Co-rearing with thrips significantly reduced active *T. urticae* abundance over 20 days, indicating that thrips can affect mite population growth under laboratory conditions. However, this reduction cannot be attributed unequivocally to direct predation. Competition for plant resources, thrips-induced changes in host-plant quality or defenses, and other plant-mediated interactions may also have contributed. By contrast, pairwise thrips co-rearing did not significantly suppress visible active-stage abundance of any thrips species, suggesting that the observed intraguild egg predation was insufficient to cause detectable population-level suppression, or that compensatory reproduction and resource availability buffered populations against these losses. Higher densities of herbivorous prey, including aphids and thrips, have been associated with a higher probability of IGP events in potato fields [20]. Plant architecture may alter encounter rates and provide refuges for prey, while prey density and the availability of alternative plant- or animal-derived foods may modify both predation and intraguild interactions. Thus, the apparent role of *T. urticae* as extraguild prey and a potential dietary subsidy for thrips should be interpreted cautiously [20,36]. Semi-field and field experiments that independently manipulate prey availability, plant condition, and habitat complexity will be needed to distinguish direct predation from resource-mediated effects and determine whether these interactions produce meaningful population suppression under natural conditions.

qPCR detection of *T. urticae* DNA in predators after mite-feeding assays and detection of thrips prey DNA after selected intraguild assays provided molecular evidence of trophic transfer. Molecular detection rates were lower than behavioral predation estimates in some assays, consistent with the expected effects of prey DNA degradation, small meal size, sampling interval, primer sensitivity, and conservative interpretation of melting-curve profiles [22,23,24,25,26,27]. DNA-based methods are most appropriately used to confirm the occurrence of trophic interactions rather than to quantify predation intensity. Moreover, secondary predation and contamination may generate false-positive detections. These results should thus be interpreted with caution.

All experiments in the present study were conducted under laboratory conditions using simplified arenas without pre-established mite colonies or accumulated silk. This is an important caveat when extrapolating findings to field or greenhouse crop systems. Under natural infestation conditions, *T. urticae* populations at high densities produce copious silken webbing that covers leaf surfaces, petioles, and stems. The webbing functions as a physical and structural anti-predator defence that has been demonstrated to reduce predation by multiple natural enemy groups. *T. urticae* webbing inhibits the movement and foraging efficiency of specialist phytoseiid mites such as *Phytoseiulus persimilis* Athias-Henriot, creating a refugium within which eggs and immatures are partially shielded from attack [37]. The web also raises the energetic cost of predator navigation within mite colonies, and patch time and predation rates by polyphagous phytoseiids such as *Typhlodromus occidentalis* Nesbitt and *Amblyseius andersoni* (Chant) decline when webbing density increases [12,37,38].

Critically, the inhibitory effect of spider mite webbing has also been directly demonstrated for thrips. Mite webbing covering eggs and the leaf surface significantly inhibited predation by *F. occidentalis* larvae in cotton, contributing to the interpretation of *F. occidentalis* as an opportunistic rather than a sustained mite predator [12]. *T. urticae* webbing protected eggs of the predatory mite *P. persimilis* from predation by *F. occidentalis* in laboratory experiments, with substantially fewer predatory mite eggs consumed when they were oviposited within mite webbing compared to a webbing-free surface [38]. Predatory thrips would encounter fewer exposed mite eggs per unit search time, effectively lowering encounter rate and reducing per-capita predation below the values measured in the present webbing-free arenas. Additionally, high mite densities are often associated with the production of chemical kairomones embedded in silk trails that mediate predator orientation; interactions between these chemical cues and thrips foraging behaviour under field conditions remain largely unknown.

Thrips predation on spider mites in field settings would be further modulated by plant architecture, mite colony distribution across the canopy, alternative food sources (pollen, plant sap), the abundance of competing natural enemies, and the susceptibility of thrips themselves to predation and parasitism [20,28,30]. Dual infestations by *T. urticae* and thrips have been shown to complicate biological control outcomes. In bean, the presence of *T. urticae* webbing reduced the efficiency of *Orius insidiosus* in suppressing *F. occidentalis*, illustrating how high mite densities can indirectly shelter thrips from their own natural enemies [39]. These interactions collectively suggest that even when thrips have the intrinsic capacity to prey on *T. urticae* eggs, as demonstrated here under simplified conditions, the realized population-level impact of thrips on mite dynamics in the field is likely to be considerably weaker and more variable. The practical contribution of vegetable thrips to spider mite suppression therefore cannot be reliably predicted from laboratory predation rates alone, and field validation under realistic mite infestation conditions is essential before incorporating thrips predation into IPM decision frameworks.

From an IPM perspective, these findings have two principal implications. First, pest thrips may contribute to suppression of spider mite populations under certain conditions, particularly through egg predation, and even modest per-capita predation rates may have demographic consequences when thrips are abundant and mite populations are low, as could occur early in the growing season [14]. Second, this predatory activity does not make thrips net beneficial to the cropping system, because the same species cause direct crop damage and may vector plant viruses. The contribution of thrips to mite suppression is strongly context-dependent; its practical relevance under field conditions with complex plant architecture, heterogeneous prey distribution, and interference by other natural enemies requires further investigation [3,4,40]. The findings support the ecological classification of vegetable thrips as context-dependent omnivores rather than simple herbivores, and suggest that their dual role should be incorporated into simulation models of pest population dynamics and integrated management strategies.

## 5. Conclusions

*Frankliniella occidentalis*, *T. palmi*, and *M. usitatus* can prey on *T. urticae*, but predation is shaped by predator identity, developmental stage, prey stage, and host-plant context. Mite eggs are the most vulnerable prey stage, and host-plant-free conditions amplify egg predation by *F. occidentalis* and *T. palmi*. Intraguild predation among thrips is comparatively limited, although the larvae of *M. usitatus* can reduce *F. occidentalis*’ egg survival. Population mixed-rearing confirms that thrips can reduce active *T. urticae* abundance over time, while qPCR provides molecular support for selected trophic links. These findings extend current understanding of thrips beyond their conventional role as herbivorous and invasive pests, highlighting their context-dependent omnivory and its relevance to integrated pest management of vegetable crop systems where invasive and native thrips and mite pests co-occur. Broad-spectrum insecticide programs targeting thrips should account for this incidental mite suppression, and thrips omnivory should be incorporated into IPM decision models.

## Figures and Tables

**Table 1:** qPCR primers used for molecular detection of prey from the predators.

Prey Species	Target Gene	Primer Name	Primer Sequence (5′→3′)	Amplicon Length (bp)	Melting Temperature (°C)	Reference
*T. urticae*	ITS1	Turti_1F	GTTTTACACTTCTTCGCCTAA	142	78.96	[32]
		Turti_1R	CACCGCTTGAAGATGTATCT			
*F. occidentalis*	ITS2	Focc1U5-F	GGTCGCTTCACCGCTTCCRCCCGTAAA	236	88.17	[33]
		Focc2D5-R	CAAAGTGCGAGAAAATAAAATGCAAACT			
*M. usitatus*	ITS2	Musi2U	GGCCTCGTGTCGTAGTGCTCTTAAATT	270	87.09	[33]
		Musi2D	ACCCGGAACTGAGGCTGGAACATAAGA			

## Data Availability

The data supporting the findings of this study are available from the corresponding author upon reasonable request.
